# Computational Studies of Molecular Materials for Unconventional Energy Conversion: The Challenge of Light Emission by Thermally Activated Delayed Fluorescence

**DOI:** 10.3390/molecules25041006

**Published:** 2020-02-24

**Authors:** Javier Sanz-Rodrigo, Yoann Olivier, Juan-Carlos Sancho-García

**Affiliations:** 1 Department of Physical Chemistry, University of Alicante, E-03080 Alicante, Spain; jsr72@alu.ua.es; 2 Unité de Chimie Physique Théorique et Structurale & Laboratoire de Physique du Solid, Namur Institute of Structured Matter, Université de Namur, B-5000 Namur, Belgium; yoann.olivier@unamur.be

**Keywords:** TADF, OLEDs, excited-states energy conversion, singlet–triplet energy gap, TD-DFT

## Abstract

In this paper we describe the mechanism of light emission through thermally activated delayed fluorescence (TADF)—a process able to ideally achieve 100% quantum efficiencies upon fully harvesting the energy of triplet excitons, and thus minimizing the energy loss of common (i.e., fluorescence and phosphorescence) luminescence processes. If successful, this technology could be exploited for the manufacture of more efficient organic light-emitting diodes (OLEDs) made of only light elements for multiple daily applications, thus contributing to the rise of a sustainable electronic industry and energy savings worldwide. Computational and theoretical studies have fostered the design of these all-organic molecular emitters by disclosing helpful structure–property relationships and/or analyzing the physical origin of this mechanism. However, as the field advances further, some limitations have also appeared, particularly affecting TD-DFT calculations, which have prompted the use of a variety of methods at the molecular scale in recent years. Herein we try to provide a guide for beginners, after summarizing the current state-of-the-art of the most employed theoretical methods focusing on the singlet–triplet energy difference, with the additional aim of motivating complementary studies revealing the stronger and weaker aspects of computational modelling for this cutting-edge technology.

## 1. Introduction: The Quest for New Energy Conversion Mechanisms for Efficient Light Emission

**Figure 1:**
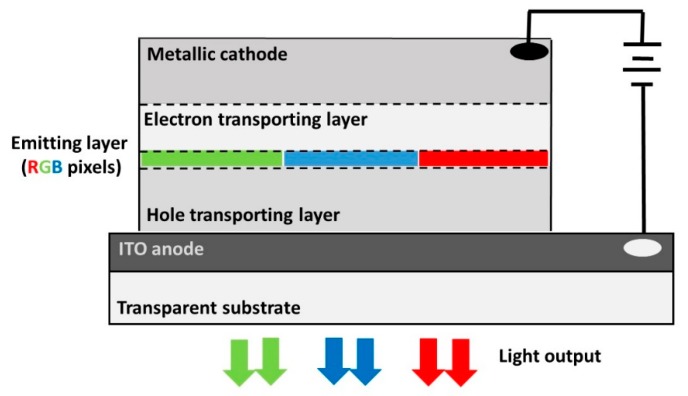
Sketch of the multi-layer architecture of a modern organic light-emitting diode (OLED) including a metallic cathode, a layer favoring the electron transport (thus blocking the hole transport), the emitting layer, a layer favoring the hole transport (thus blocking the electron transport), the indium tin oxide (ITO) anode, and the transparent substrate. In an OLED device, the light-emitting active molecules are excited after the electron–hole recombination (i.e., exciton formation) from the cathode and the anode, with associated light emission when returning to the ground state.

The development of emitter materials for the new generation of organic light-emitting diodes (OLEDs) [1] currently faces a few key challenges, such as the search for more efficient, bright, and long-lasting blue color emitters or the fine-tuning of low-cost large-scale fabrication methods such as ink-jet printing and related techniques. Despite the multi-layer (and complex) architecture of modern OLEDs (see Figure 1), the molecular emitters constituting the active layer still critically determine the efficiency of the whole electroluminescence process taking place within the device. The subpixels used to generate the basic colors (red, green, and blue) should all have similar performance (i.e., brightness) and lifetime (i.e., avoid degradation and burn-in effects) to increase resolution and user experience. Previously exploited light-emission mechanisms—fluorescence and phosphorescence—have a limited efficiency (i.e., 25% in the case of fluorescence) or a lack of competitive phosphorescent deep-blue emitting molecules [2].

On the other hand, thermally activated delayed fluorescence (TADF) [3,4,5], also known as E-type fluorescence in the past, can help to achieve these ambitious goals thanks to the combination of pure organic materials, without relying on heavy metals (in contrast to phosphorescence). It has efficient and long-lifetime performance, which could thus pave the way towards its ultimate commercialization. In fact, several companies (e.g., Kyulux, Cynora, LG Display, Novaled, Wisechip, Idemitsu Kosan, Toray Industries, UDC, Noctiluca, etc.) are investing time and effort to commercializing this new family of compounds. The year 2019 also notably marked the 10-year anniversary since the first demonstration of TADF OLEDs, but it seems there is still work to be done to fully reach this longstanding goal [6,7].

**Figure 2:**
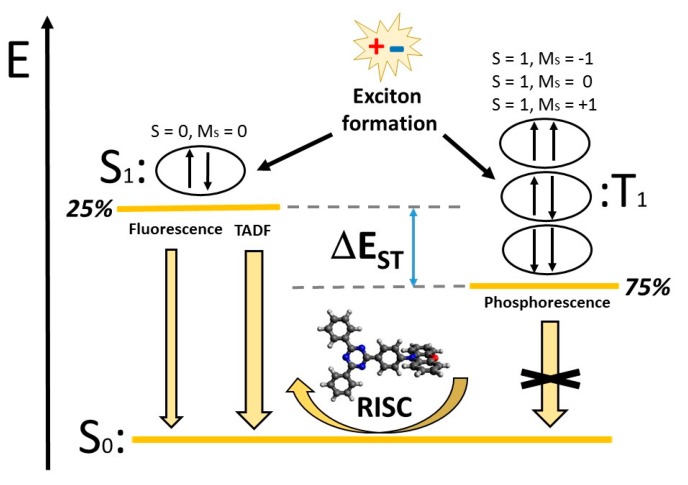
Sketch of the energy levels for the ground state (S_0_) and lowest excited states (S_1_ and T_1_) of different multiplicity, involved in the thermally activated delayed fluorescence (TADF) mechanism after the exciton formation, and the corresponding reverse intersystem crossing mechanism (RISC) process from the T_1_ to the S_1_ states compared to fluorescence (emission from S_1_) and phosphorescence (emission from T_1_). Note the ideal energy up-conversion from the triply-degenerated T_1_ to the S_1_ state for sufficiently small ΔE_ST_ values.

The idea behind a more efficient molecular emitter embedded into a functional matrix and acting as the active layer of a built-in TADF-based OLED relies on harvesting 100% of the excitons formed upon the electrical excitation, in contrast to former light-emitting mechanisms such as fluorescence or phosphorescence whose internal quantum efficiencies (IQEs) were limited a priori as said previously. In the TADF mechanism, 75% of triplet excitons could ideally be recovered through a reverse intersystem crossing mechanism (RISC) due to quasi-resonant lowest singlet and triplet excited states, S_1_ and T_1_ respectively (see Figure 2). Note that the singlet–triplet energy difference (ΔE_ST_) in most conjugated molecules and polymers is around 0.5–1.0 eV [8], thus precluding an efficient RISC in the absence of other driving factors. When the energy difference ΔE_ST_ remains around 0.1–0.2 eV, one could therefore envision an RISC rate within the semi-classical Marcus formalism [9], $k_{RISC}\propto V_{SOC}^{2}\;e^{-\left(\frac{{\left(\lambda +\Delta E_{ST}\right)}^{2}}{4\lambda k_{B}T}\right)}$, fast enough to achieve a delayed fluorescence in addition to the standard fluorescence from the remaining 25% of singlet excitons. We are also aware of other technical requirements, such as: short excited-state lifetime to avoid non-radiative decay and thus maximize the photoluminescence quantum yields (PLQYs), appropriate color CIE coordinates and color purity, etc. However, in the following sections we focus on the basic aspects for the molecular modelling, at the molecular scale, related to energy conversion [10].

## 2. Current Achievements and Challenges for Theoretical Methods at the Molecular Scale

However, the search for novel (purely organic) molecular materials with close in energy S_1_ and T_1_ states is not exempted from difficulties due to the myriad possible candidates to be explored. Fortunately, the computational design of novel conjugated organic materials has significantly contributed to the field, first applying a variety of modelling techniques for the fast screening of the compounds, and then rationalizing the physical origin of small ΔE_ST_ together with other competitive factors such as the impact of the spin–orbit coupling (SOC or $V_{SOC}$). Despite significant advances in the study and influence of SOC [11,12,13], which also contributes to the RISC rate as $k_{RISC}\propto V_{SOC}^{2}$, in the following we concentrate on the former ΔE_ST_ quantity for simplicity.

**Figure 3:**
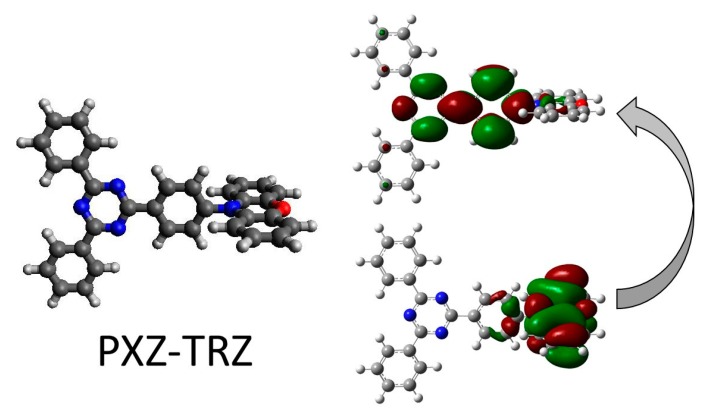
**Left**: Chemical structure of 2-phenoxazine-4,6-diphenyl-1,3,5-triazine (PXZ-TRZ), a commercially available green emitter that is widely used for TADF and exhibits external quantum efficiencies up to 21% [16]. **Right**: Isocontour plots of the HOMO (top) and LUMO (bottom) orbitals, with the green and red color of the lobes denoting their different sign. An excited state—S_1_ or T_1_—created mostly by a HOMO-to-LUMO one-electron promotion will involve some degree of intramolecular charge-transfer character due to the spatial localization of both orbitals on different molecular moieties, D and A, respectively.

In the simplest possible and uncorrelated (Hartree–Fock) case, supposing that both S_1_ and T_1_ states are formed exclusively by a one-electron excitation (if this is not formally the case, one can always use a natural transition orbital representation or the attachment/detachment formalism) from the highest occupied molecular orbital (HOMO or simply H) to the lowest unoccupied molecular orbital (LUMO or simply L), one easily infers that ΔE_ST_ roughly relates to the exchange energy (i.e., the value of the double integral $\int \;\int \;\phi_{H}^{*}\left(r\right)\phi_{L}\left(r\right)\frac{1}{\left|r-r^{\prime }\right|}\phi_{H}\left(r^{\prime }\right)\phi_{L}^{*}\left(r^{\prime }\right)drdr^{\prime }$), which should thus be minimized for any set of molecules tackled. Previous works on donor–acceptor (DA) conjugated systems found that a poor overlap between the involved HOMO ($\phi_{H}$) and LUMO ($\phi_{L}$) orbitals, when they interact only through their exponential tails, would lead to sufficiently small ΔE_ST_ values [14,15]. Therefore, computational calculations offered, from the very beginning and mostly using time-dependent density-functional theory (TD-DFT) methods, an intuitive and cost-effective tool to look at the spatial distribution of those orbitals as a simple indicator for the screening and choice of suitable candidates (Figure 3).

Beyond this qualitative picture, the prediction of the energy of the S_1_ and T_1_ states, E(S_1_) and E(T_1_) respectively, is obviously a key target to achieve in order to accurately assess the magnitude ΔE_ST_ = E(S_1_) − E(T_1_), and eventually the energy of any other S_n_ and T_n_ states defining the full excited-states energy manifold. As simple as it seems, this is still a challenging goal [17]. The use of the cost-effective TD-DFT method, independently of the type of calculation done (i.e., the underlying selected exchange-correlation functional E_xc_[ρ]), has revealed a set of serious shortcomings affecting the reliability and reproducibility of the computational results for TADF applications. Note that the common deviations found in earlier and benchmark applications to large sets of organic materials (TD-DFT is generally agreed to provide an averaged mean absolute deviation of 0.3 eV, roughly speaking, between calculated and experimental absorption or emission low-lying energies, with all due caution about specific cases [18,19]) could be the reason to choose—or discard—a TADF-based molecule based on the sole criteria of providing a low ΔE_ST_ value. Using exciton properties as reference properties for benchmarking theoretical methods could also possibly be exploited [20]. Among the known shortcomings, we remark: (i) the difference in quality between E(S_1_) and E(T_1_) calculations, with unbalanced errors for both quantities, as well as with respect to the historically used “ΔSCF” method to predict the energy of the T_1_ state; (ii) the neglect of pure double-electron excitations using conventional TD-DFT methods; and (iii) the relatively large spread of the results using different exchange-correlation functionals or, in other words, the possibility of having system-dependent results upon the functional choice.

This situation is not surprising within the context of correctly predicting intramolecular charge-transfer (ICT) excitations, since for molecular materials this has always been a major challenge [21]. Some of these shortcomings were partly remedied with the recent use of tuned exchange-correlation functionals based on the matching between theoretically predicted and experimentally available one-electron (i.e., the energy of the H and/or L orbitals, $\epsilon_{H}$ and $\epsilon_{L}$) and many-electron (i.e., the energy of the corresponding ionized states) energies for each of the molecules studied—a computational protocol later extended directly to the matching of theoretical and experimental E(S_1_) and/or E(T_1_) energies. Invoking again the concept of DA compounds at their infinite separation, it is easy to see that the excitation energy will be given by IP(D) – EA(A), where IP is the ionization potential of the donor fragment and EA is the electron affinity of the acceptor fragment. This suggests the aforesaid tuning at intermediate separations: $\epsilon_{H}\approx$ IP(D) and $\epsilon_{L}\approx$ EA(A). Note also that: (i) the mentioned tuning is possible thanks to the flexibility of the ingredients (and their weights) entering into modern expressions for exchange-correlation functionals (e.g., the range-separation parameter); and (ii) a system-specific tuning was also previously explored in the context of TADF adjusting the fraction of exact-like exchange (i.e., exact exchange energy but calculated with the self-consistently obtained Kohn–Sham orbitals) of the more classical hybrid functionals. These last efforts do not solve other issues, such as the *n*-tuple (*n* being double or higher) excitations, besides the increase of the computational effort and the lack of transferability of the results. However, they have definitively contributed to the estimation, for instance, of the influence of polarization effects after calculating the difference in E(S_1_) and E(T_1_) energies from gas phase to polarizable environments [22,23].

## 3. Understanding the Grounds of a TD-DFT Calculation

Current implementations of TD-DFT in most codes make use of the linear-response formalism, in which for pure (non-hybrid) density functionals the exact excitation energies $\hbar \omega_{n}$ are obtained from the eigenvalues of the matrix $M_{ia\sigma ,jb\tau }={\left(\epsilon_{a\sigma }-\epsilon_{i\tau }\right)}^{2}\delta_{ij}\delta_{ab}\delta_{\sigma \tau }+2\sqrt{\epsilon_{a\sigma }-\epsilon_{i\sigma }}\;\sqrt{\epsilon_{b\tau }-\epsilon_{j\tau }}\;K_{ia\sigma ,jb\tau }\left(\omega \right)$, where the indices *ij* (*ab*) refer to occupied (virtual) orbitals and στ are spin indices. Interestingly, the contribution from the term $K_{ia\sigma ,jb\tau }\left(\omega \right)$ ideally includes the many-body and frequency-dependent effects beyond one-electron energy orbital differences. This term splits into two contributions, $K_{ia\sigma ,jb\tau }\left(\omega \right)=\int \;\int \;\phi_{i\sigma }^{*}\left(r\right)\phi_{a\tau }\left(r\right)\left(\frac{1}{\left|r-r^{\prime }\right|}+f_{xc}\left(r,r^{\prime },\;\omega \right)\right)\phi_{j\tau }^{*}\left(r^{\prime }\right)\phi_{b\tau }\left(r^{\prime }\right)drdr^{\prime },$ with the second contribution depending on the second functional derivative (i.e., the kernel) of the exchange-correlation functional, $f_{xc}\left(r,t;r^{\prime },\;t^{\prime }\right)=\frac{\delta^{2}E_{xc,\sigma }(\left[\rho_{\sigma }\right];r,t)}{\delta^{2}\rho_{\sigma }\left(r^{\prime },t^{\prime }\right)}$, which is by definition exact and non-local in space (i.e., dependence on r and $r^{\prime }$) and non-local in time (i.e., dependence on t and $t^{\prime }$) to incorporate the ω-dependency. We would like to emphasize the importance of this term in TD-DFT, especially in TADF, which basically would turn (approximate) one-electron into (exact) many-electron excitations.

However, several approximations are used in practice to calculate, for example, the E(S_1_) and E(T_1_) energies through the linear-response TD-DFT formalism, again independent of the expression selected for E_xc_[ρ]:

(i) All excited-state calculations are done converging first the ground-state eigenvalues and orbitals, and then using those orbitals and the associated density to calculate all TD-DFT-related magnitudes, which thus precludes the incorporation of an explicit dependence on time in the kernel $f_{xc}$—a fact known as the adiabatic approximation;
(ii) The use of complex mathematical expressions for the integrand of modern semilocal E_xc_[ρ] functionals (i.e., GGA or meta-GGA) makes it difficult to obtain their second functional derivative analytically, and often the simplest expression known as the local density approximation (LDA) is used instead $\left[f\left(r,r^{\prime },\;\omega \right)\approx f_{xc}^{LDA}\left(r\right)\delta \left(r-r^{\prime }\right)\right]$ to speed up the calculations. The good performance of the adiabatic LDA (ALDA) approximation in finite systems has discouraged the development of more elaborate ways to incorporate the time dependence [24].
(iii) Again, the use of a semilocal E_xc_[ρ] functional, depending only on ρ(r) and its successive derivatives, precludes the incorporation of an explicit spatial dependence in $f_{xc}$.

Briefly speaking, neither the temporal nor the spatial memory is contained in $f_{xc}$, thus keeping an oversimplified form. Note that the use of a tuned range-separated functional or the Tamm–Dancoff approximation (TDA), for instance, would not overcome these common drawbacks of TD-DFT calculations, although they would slightly improve the agreement with respect to experimental results, especially for triplet excitations bearing a low-to-medium degree of ICT excitations.

These shortcomings do not strongly affect the performance of TD-DFT for non-CT excitations, i.e. for those excitation being mostly local, beyond that average accuracy obtained in benchmark studies, but the role of the $\int \;\int \;\phi_{i\sigma }^{*}\left(r\right)\phi_{a\tau }\left(r\right)\;f_{xc}\left(r,r^{\prime },\omega \right)\phi_{j\tau }^{*}\left(r^{\prime }\right)\phi_{b\tau }\left(r^{\prime }\right)drdr^{\prime }$ integral is normally underestimated in TADF applications. Solving exactly this problem is far from being trivial: the small but non-vanishing overlap between the involved orbitals giving rise to the S_1_ and T_1_ states would need to be incorporated through the spatial non-locality of the kernel $f_{xc}$, which is not possible in current formulations beyond some exceptions, deteriorating thus the accuracy for charge transfer (CT) excitations. In the limit of infinitely separated D and A fragments, $K_{ia\sigma ,jb\tau }\left(\omega \right)$ will vanish and thus the excitation energy will be wrongly given by eigenvalue difference, but at intermediate separations this contribution can be substantially modified (decreasing or increasing) each of the excited-state energies with respect to the first contribution $\int \;\int \;\phi_{i\sigma }^{*}\left(r\right)\phi_{a\tau }\left(r\right)\left(\frac{1}{\left|r-r^{\prime }\right|}\right)\phi_{j\tau }^{*}\left(r^{\prime }\right)\phi_{b\tau }\left(r^{\prime }\right)drdr^{\prime }$.

Finally, we also discuss here the importance of double excitations in calculations for TADF studies. This issue has also been previously observed for conjugated polyenes and biochromophores [25,26], but it has been largely ignored in the TADF field. However, recent synthetic advances have revealed how TD-DFT could not even qualitatively predict the ΔE_ST_ value (i.e., errors up to 0.5 eV) for a set of particularly interesting TADF compounds known as multi-resonant TADF emitters [27,28,29], bearing some degree of double-excitation character in addition to other electronic effects. A double-excited state is formed by simultaneously promoting two electrons from occupied to virtual orbitals. Whereas the correct description of these states, be them ground states or excited states, is clearly based on correlation effects, the full wavefunction is given by $|\Psi \rangle =c_{0}|\Psi_{0}\rangle +c_{i}^{a}|\Psi_{i}^{a}\rangle +c_{ij}^{ab}|\Psi_{ij}^{ab}\rangle +\cdots$, where $|\Psi_{i}^{a}\rangle$ is a singly-excited determinant, $|\Psi_{ij}^{ab}\rangle$ is a doubly-excited determinant, etc., and the corresponding energy by the sum of uncorrelated (E_0_) and correlation energy, E = E_0_ + E_c_, how these states might be described by linear-response TD-DFT is still a matter for further investigation.

## 4. Beyond a TD-DFT Treatment of TADF

The underlying trade-off between accuracy and computational cost should always be considered for any theoretical application with predictive power: real-life molecules are often constituted by tens or even hundreds of atoms, which helps to explain the great initial success of TD-DFT for the field of TADF, with only very limited applications of other ab initio methods to date. After having enumerated the issues to be solved with TD-DFT calculations for TADF compounds, which however does not preclude its widespread use as a complementary tool, it is now easier to understand why cost-effective solutions for excited states based on ab initio methods might emerge as a reliable alternative for difficult cases.

Therefore, since the spin-component-scaled (SCS-)CC2 method has recently been successfully applied to TADF compounds [30] for which TD-DFT calculations failed, in the following we revisit its main features. The method is a variant of the coupled-cluster (CC) general theory in which the ground-state energy is obtained by $E_{CC}=\langle \Psi_{0}\left|e^{-\hat{T}}\hat{H}e^{\hat{T}}\right|\Psi_{0}\rangle$, where $|\Psi_{0}\rangle$ is the ground-state reference (i.e., Hartree–Fock) wavefunction. The cluster operator $e^{\hat{T}}$ can be expanded as a Taylor series as $1+\hat{T}+\frac{1}{2!}\;\hat{T_{}^{2}}+\frac{1}{3!}\;\hat{T_{}^{3}}+\cdots$, with $\hat{T}=\hat{T_{1}}+\hat{T_{2}}+\cdots +\hat{T_{n}}$, where $\hat{T_{1}}$ is the operator for all single excitations, $\hat{T_{2}}$ is the operator for all double excitations, and so forth. In the formalism of 2nd-quantization, these excitation operators are ${\hat{T}}_{1}=\underset{ia}{\sum }t_{a}^{i}\;\hat{a^{a}}\;\hat{a_{i}}$, ${\hat{T}}_{2}=\frac{1}{4}\underset{ia,jb}{\sum }t_{ab}^{ij}\;\hat{a^{a}}\;\hat{a^{b}}\;\hat{a_{i}}\;\hat{a_{j}}$, etc., where *ij* (*ab*) denote occupied (unoccupied) hole–particle orbital states and $\hat{a^{a}}$ ($\hat{a_{i}}$) the corresponding creation (annihilation) operators. The different truncations will define the corresponding CC level: CCSD (with only single and double excitations, $\hat{T}=\hat{T_{1}}+\hat{T_{2}}$), CCSDT (with single, double, and triple excitations, $\hat{T}=\hat{T_{1}}+\hat{T_{2}}+\hat{T_{3}}$), etc. Even in its simple CCSD formulation, the exponential operator thus becomes $e^{\hat{T}}=1+\hat{T_{1}}+\hat{T_{2}}+\frac{1}{2!}\;\hat{T_{1}^{2}}+\frac{1}{2!}\;\hat{T_{1}}\;{\hat{T}}_{2}+\frac{1}{2!}\;\hat{T_{2}^{2}}+\cdots$ and also includes excitations of the form $\hat{T_{1}}\;{\hat{T}}_{2}$ (disconnected triples) or $\hat{T_{2}^{2}}$ (disconnected quadruples).

The CCSD energy is thus given by $E_{CCSD}=\langle \Psi_{HF}\left|\hat{H}e^{\hat{T_{1}}+\hat{T_{2}}}\right|\;\Psi_{HF}\rangle ,$ with the corresponding amplitudes obtained from the equations $\langle \mu_{i}|e^{\hat{-T_{1}}-\hat{T_{2}}}\hat{H}e^{\hat{T_{1}}+\hat{T_{2}}}|\Psi_{HF}\rangle$, where $\mu_{i}\;\left(i=1,2\right)$ denotes the single and double excitations manifold. If the CCSD equations are approximated, the singles are retained but the doubles are approximated to be correct up to second-order only, and one obtains the CC2 method [31]. The energy thus becomes $E_{CC2}=E_{HF}+\overset{ab}{\underset{ij}{\sum }}\left[t_{ab}^{ij}+t_{a}^{i}t_{b}^{j}\right]\left[2\left(ia|jb\right)-\left(ja|ib\right)\right]$, with the cluster amplitudes $t_{a}^{i}$ and $t_{ab}^{ij}$ obtained iteratively. For instance, for the case of the sets of all singly excited determinants $\mu_{1}$, the equation to be solved is $\Omega \left(\mu_{1}\right)=\langle \mu_{1}\left|\hat{H}+\left[\hat{H},\hat{T_{2}}\right]\right|\Psi_{0}\rangle =0$, from which we can easily observe how single- and double-excitations are coupled and naturally enters into the CC2 treatment. Next, the introduction of different scaling factors for the same-spin and opposite-spin contributions to the correlation energy gives rise to the SCS variant, with a hopefully better performance after mimicking those effects from higher-order excitations [32,33]. The excitation energies are also obtained by linear-response theory as derivatives with respect to the cluster amplitudes (e.g., $\frac{\partial \Omega \left(\mu_{i}\right)}{\partial t_{a}^{i}}$), but at a cost scaling as $O\left(N^{5}\right)$ with respect to the system size N and thus considerably higher than that of TD-DFT scaling as $O\left(N^{3-4}\right)$.

Note that not only (SCS-)CC2 can provide considerably accurate excitation energies for ICT and double-excited states of real-life TADF compounds, but other existing methods such as CIS(D) [34], configuration interactions with single and perturbatively approximated double excitations (SCS-)ADC(2) [35], algebraic diagrammatic construction at second-order, NEVPT2 [36], N-electron valence perturbation theory at second-order, EOM-CCSD [37], equation-of-motion coupled clusters with single and double excitations, CASPT2 [38], complete active space second-order perturbation theory, DFT-MRCI [39], density-functional theory coupled with multi-reference configuration interactions, BSE@GW [40], Bethe–Salpeter equation with Green’s functions, etc. could also be tested on the quest for the most accurate description of these systems.

## 5. Case Study: 2CzPN and DABNA-1/TABNA Compounds

**Figure 4:**
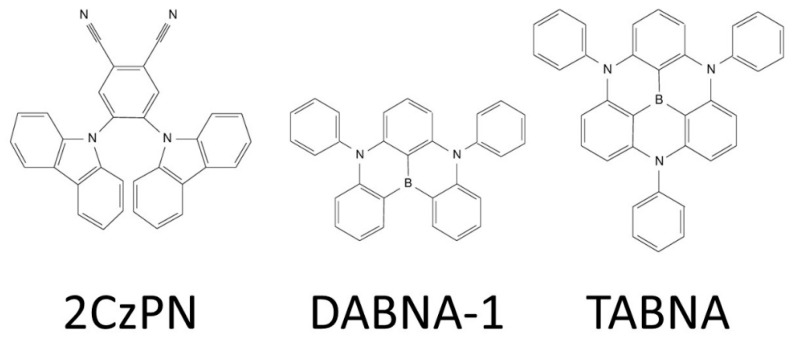
Chemical structure (from left to right) of the 1,2-bis(carbazol-9-yl)-4,5-dicyanobenzene (2CzPN) and the DABNA-1/TABNA triangulene derivatives.

In the following we illustrate the previous arguments by applying TD-DFT and a pair of ab initio methods (SCS-CC2 and NEVPT2) to a set of prototype TADF molecular materials: (i) the 1,2-bis(carbazol-9-yl)-4,5-dicyanobenzene (2CzPN) molecule; and (ii) a pair of B-centered N-substituted triangulene derivatives (Figure 4). The 2CzPN system is a commercially available highly efficient blue-sky emitter, with two electron-donating carbazolyl moieties attached to the electron-withdrawing dicyanobenzene ring. On the other hand, the latter systems (dubbed as DABNA-1 and TABNA in the literature) have recently been proposed as new candidates providing narrow emission spectra, thus maximizing color purity and having high PLQY, thus minimizing non-radiative decays thanks to their particular (multi-resonant) electronic structure [27,28,29]. Contrarily to former prototypical molecules, these triangulene-engineered compounds are relatively rigid (which contributes to low reorganization energies, and thus negligible Stokes shift and narrow emission spectra) also keeping low ΔE_ST_ values, which have fostered recent investigations to shed light about the physical origin of this unexpected behavior [30,41,42].

Table 1 shows the energy values for the set of molecules selected, as calculated by different methods with the sufficiently large def2-TZVP basis set. Note that the experimental E(S_1_) and E(T_1_) energies are taken from solution using polar solvents and/or thin-films experiment, and could be thus affected by strong solvatochromic or polarization matrix effects due to its CT nature [43]. Therefore, as stated previously, we will focus on the trends affecting the ΔE_ST_ values. Note also that: (i) the multi-configurational NEVPT2 method relies on the complete active space self-consistent field (CASSCF) method, introducing both non-dynamical and dynamical correlation effects; (ii) both the CASSCF and NEVPT2 methods need the choice of a reasonable active space, *N* electrons distributed among *M* orbitals, which is done here based on a calculated fractional occupation of orbitals ((8,8) for 2CzPN and (10,10) for DABNA-1 and TABNA). The fractional occupation (*f_i_*) of orbitals is obtained by the finite-temperature DFT method [44,45]. Briefly, the fractional occupation is induced by minimizing the Gibbs electronic free energy (G_el_ = E_el_ − T_el_ S_el_) of the system at a fictitious pseudo-temperature (i.e., electronic) called T_el_, with the *f_i_* values obtained by a smeared distribution around the Fermi level. We used as a cutoff to define the active space of those orbitals with a fractional occupation *f_i_* < 0.98 and *f_i_* > 0.02, and thus appreciably populated. Furthermore, the implementation of the state-averaged CASSCF and NEVPT2 methods also needs to fix the number of roots of each multiplicity (S_n_, T_n_) to be simultaneously calculated, which was chosen here as (8,8) after looking at the convergence of the results with respect to that technicality.

For 2CzPN, there is a close agreement between TDA-PBE0 and the more sophisticated SCS-CC2 and NEVPT2 methods, as well as with respect to the experimental result—a fact that was also documented before [30]. However, for both DABNA-1 and TABNA molecules, TDA-PBE0 provides a relatively large ΔE_ST_ value of around 0.6 eV, and is thus incompatible in principle with a TADF mechanism, and far from the experimental results. On the other hand, SCS-CC2 and NEVPT2 methods give considerably lower ΔE_ST_ values (approximately between 0.1–0.2 eV), and are thus very close to the experimental estimates. In fact, in a previous publication by some of the authors [30], we specifically compared various theoretical levels for the DABNA-1 compound (CC2, SCS-CC2, and STEOM-CCSD, with ΔE_ST_ values between 0.12 and 0.17 eV) with an experimental result of 0.14 eV. Concerning oscillator strength values, all methods give non-vanishing values, and are thus compatible with the reasonable experimentally observed PLQY.

We will start rationalizing these results by inspecting first the double-excitation nature of the S_0_, S_1_, and T_1_ excited state formed in all the compounds. This can be done through analyzing the CAS wavefunction, in which the NEVPT2 correction is based, as stated previously. The CAS wavefunction can be expressed as an expansion of simply-, doubly-, triply-substituted, etc. Slater determinants, with *M* being the subset of those selected active orbitals, as: $|\Psi_{CAS}\rangle =\underset{M}{\sum }C_{M}|\Psi_{M}\rangle =c_{0}|\Psi_{0}\rangle +\underset{i}{\sum }c_{i}^{a}|\Psi_{i}^{a}\rangle +\underset{ij}{\sum }c_{ij}^{ab}|\Psi_{ij}^{ab}\rangle +\underset{ijk}{\sum }c_{ijk}^{abc}|\Psi_{ijk}^{abc}\rangle +\cdots$ weighted by the corresponding coefficients $c_{i}^{a},\;c_{ij}^{ab},\;c_{ijk}^{abc},\;\ldots$ The relative *n*-tuple (n≥2) coefficients ($c_{ij\ldots }^{ab\ldots }$) amount to 1.0%, 8.5%, and 5.4% (0.7%, 3.3%, and 1.9%) for the S_1_ (T_1_) excited state of 2CzPN, DABNA-1, and TABNA, respectively, with respect to the sum of all the coefficients. Note that this difference between 2CzPn and DABNA-1/TABNA compounds is also seen at the S_0_ ground state: 0.0%, 3.8%, and 1.2%, respectively. These results show the negligible (significant) impact of mostly double (but also higher) excitations for 2CzPN (DABNA-1 and TABNA)—an effect that is currently neglected by the linear-response formulation of the TD-DFT method and introduced (at least partly) by both NEVPT2 and SCS-CC2 through the dynamical correlation contribution. Note also the general difficulties in dealing with marked double excitations for real-world compounds, which is still a matter of debate within the theoretical community [46].

The importance of these findings is worth note. First, the correct description of the singlet–triplet energy difference in standard (e.g., 2CzPN) and complex (e.g., DABNA-1/TABNA) TADF compounds is a more complex issue than simply looking at the HOMO–LUMO (uncorrelated and/or kernel-independent) exchange energy, and requires the inclusion of correlation effects at high orders. This is approximately done by the CC2 method at first and second order, and the SCS parameterization seems to efficiently include (at least partly) the missing correlation energy. On the other hand, the use of multi-configurational methods (e.g., NEVPT2) also allows both components of the correlation energy, non-dynamical and dynamical, to be dealt with in a balanced way. However, we also need to aware of the influence of the active space and the number of roots (S_n_, T_n_) demanded in the final NEVPT2 results.

## 6. Summary and Prospects

The rise in the massive deployment of OLED technologies for displays has fostered the search of new light-emitting mechanisms (e.g., TADF) to improve the device efficiency beyond the 25% fluorescence limit. The expected outcomes are two-fold: the improvement of energy efficiency as well as an increase in the resolution of OLED displays. Academic and industrial research has significantly contributed to the discovery and improvement of molecular materials for red, green, and blue pixels, although compounds for the latter are still facing few challenges. Fortunately, the theoretical modelling in recent years has guided the understanding and screening of potential molecular candidates, not only by a fast inspection of the shape and location of the frontier molecular orbital, but also by allowing the calculation of the singlet–triplet energy difference and other related energy magnitudes. However, this last target is very challenging for TADF compounds, and often demands the application of a variety of theoretical methods beyond standard TD-DFT applications. This last point is illustrated here by selecting a set of real-world molecules differing considerably in their electronic structure, despite being widely used as TADF emitters, and showing how electronic structure methodologies such as SCS-CC2 or NEVPT2 (or related) can contribute to reach chemically accurate results, providing useful insights for molecular design.

## Figures and Tables

**Table 1:** Vertical excitation energies (in eV) of the lowest singlet and triplet states and their energy difference. The oscillator strength (f_osc_) values are also included.

Compound	Method	E(S_1_)	E(T_1_)	ΔE_ST_	f_osc_ (S_0_ ← S_1_)
2CzPN	TDA-PBE0	3.01	2.67	0.34	0.08
	SCS-CC2 ^a^	3.65	3.30	0.35	0.12
	NEVPT2	3.33	3.02	0.32	0.20
	Experimental ^b^	2.94	2.63	0.31	--
DABNA-1	TDA-PBE0	3.25	2.69	0.56	0.25
	SCS-CC2 ^a^	3.25	3.10	0.15	0.31
	NEVPT2	3.04	2.95	0.09	0.21
	Experimental ^c^	2.74	2.59	0.14	--
TABNA	TDA-PBE0	3.69	3.12	0.57	0.12
	SCS-CC2 ^a^	3.67	3.50	0.17	0.13
	NEVPT2	3.16	2.99	0.18	0.32
	Experimental ^d^	3.11	2.90	0.21	--

^a^ Taken from Ref. [30]. ^b^ Data from experiments done in toluene. ^c^ Data from experiments done in EtOH [27]. ^d^ Data from experiments done in a PMMA film, 1 wt% [27].
